# Treatment providers' perspectives on a gender-responsive approach in alcohol and drug treatment for women in Belgium

**DOI:** 10.3389/fpsyt.2022.941384

**Published:** 2022-08-30

**Authors:** Julie Schamp, Wouter Vanderplasschen, Florien Meulewaeter

**Affiliations:** ^1^Department of Social Educational Care Work, University of Applied Sciences and Arts, Ghent, Belgium; ^2^Department of Special Needs Education, Faculty of Psychology and Educational Sciences, Ghent University, Ghent, Belgium

**Keywords:** alcohol and drug treatment, substance use, gender, women, gender-transformative, prevention, gender-responsive, trauma-informed care

## Abstract

**Background:**

Gender inequity is a pervasive challenge to health equity on a global scale, and research shows the impact of sex and gender on substance use regarding for example epidemiology, treatment needs, treatment admission and treatment outcomes. The gender-transformative approach to action and health indicates that health interventions may maintain, exacerbate or reduce gender-related health inequalities, depending on the degree and quality of gender-responsiveness within the programme or policy. However, research shows a lack of gender-responsive initiatives in the alcohol and drug addiction field.

**Aims:**

The purpose of this study is to explore in depth how alcohol and drug treatment can be made more sensitive to female users' treatment needs from the perspective of service providers. Consequently, study findings can inform the development of gender-responsive treatment options and aid to a deeper understanding of how these trends are designated on the continuum of approaches to action and health in the alcohol and drug field.

**Methods:**

Four focus groups were organized across different regions in Belgium with a total of 43 participants, including service providers, policy makers and women who use(d) drugs.

**Results:**

The perspective of the participants on substance use prevention and treatment for female users incorporates some crucial gender-specific and gender-transformative features. Next to implementing mother-child options, a holistic approach, experts by experience and empowering women in treatment, professionals report the relevance of awareness raising campaigns targeting all levels and sectors in society. Also, recurring attention was given to the role of men in the narratives of female users.

**Conclusion:**

Study findings show that the field of alcohol and drug prevention and treatment is being looked at through the lens of gender-responsiveness. However, to achieve improvement in the lives of both women and men, and hence creating more equal chances and opportunities in substance abuse treatment, the gender-transformative approach in addiction care needs to be further explored, criticized and established in practice and future research.

## Introduction

Gender inequity is a pervasive challenge to health equity on a global scale (1–3). Over the last two decades gender has been increasingly recognized by the global community, not as a static phenomenon, but rather a social determinant of health (4). Moreover, both sex and gender are fundamental determinants of health (5). While biological sex may differentially affect health conditions (6, 7), gender socialization and power relations generate many health challenges for all genders that change over time, giving further support to the understanding of gender as a dynamic construct (8, 9).

It is established that individual characteristics and treatment approaches can differentially affect outcomes by gender (10, 11). Recent international research shows substantial progress in our understanding of the influence of gender on, for example, the epidemiology of substance use (12, 13), and its relationship to treatment needs, treatment admission and utilization, and treatment outcomes and effectiveness (14–16).

Research has shown that internal, structural and external factors may influence help-seeking behavior and hamper treatment entry among female substance users (17–19). In addition to external and structural barriers, internal barriers such as shame, guilt and denial of problem substance use are associated with gender violation (20, 21). External barriers that can prevent women from accessing treatment are: limited treatment availability and treatment cost, absence of referral by general practitioners, and a lack of perceived treatment options and identification of services. Although child custody concerns are among the most frequently reported barriers to treatment among female substance users with children (22–25), retaining or regaining custody may also serve as leverage to treatment adherence (26). Similarly, mothers with past child custody loss may internalize this as a barrier to help-seeking, as they feel like they have nothing left to fight for (27), or it may serve as a facilitator for treatment in order to regain custody. The issue of child custody illustrates that help-seeking decisions are multi-layered, dynamic and ambiguous in relation to the meaning-making of women, which impacts help-seeking behavior in different ways (19). Furthermore, external barriers, such as judgemental attitudes of service providers and social stigma, can lead to self-stigma (28). Self-stigmatization results from the internalization of negative stereotypes associated with public stigmatization of persons with substance use disorders (29). Affected individuals will then exclude themselves from public life and be motivated to continue to consume in order to forget, set aside, or reduce the negative feelings arising from their shame (30). Moreover, research shows social stigma to be an even greater barrier to treatment for women than for men (31, 32). Also, previous research on health services generate a range of findings on the organizational characteristics that either facilitate or inhibit treatment entry for women with substance use disorders (33, 34). Thus, women and mothers who use drugs experience a number of additional barriers to treatment (35), including strong maternal and family responsibilities, lack of childcare while being in treatment, scarce economic resources, lack of support from a social network or partner, and greater social stigma.

**Figure 1:**
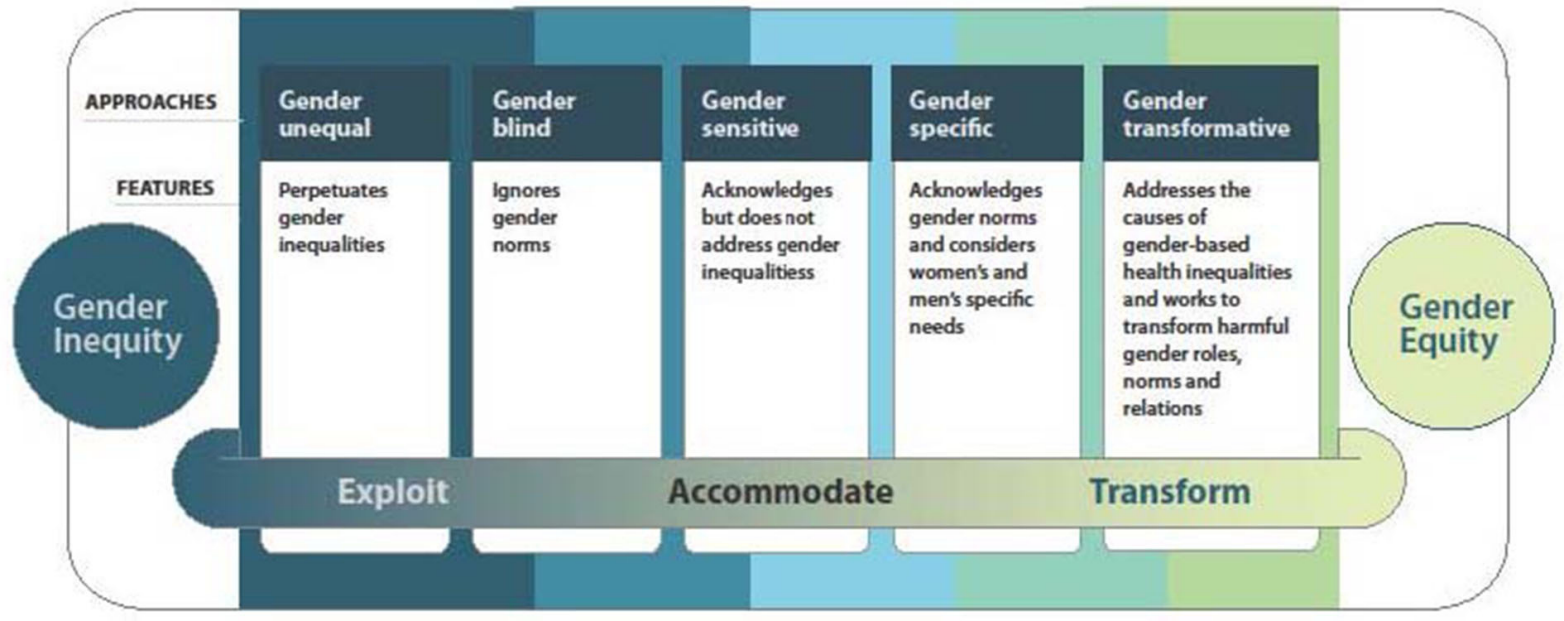
A continuum of approaches to action on gender and health. Inspired by remarks by Geeta Rao Gupta, Ph.D, Director, International Center for Research on Women (ICRW) during her plenary address at the XIIIth International Aids Conference, Durban, South Africa, 12 July 2000: “To effectively address the intersection between HIV/AIDS and gender and sexuality requires that interactions should, at the very least, not reinforce damaging gender and sexual stereotypes” [see also (40)].

In order to address these barriers, and attain more gender equity in addiction care, research shows the need for more gender-responsiveness in health programmes and interventions. Women's empowerment is an important determinant to achieve gender equality and needs to be incorporated into service delivery. Enhancing women's empowerment is a process of awareness-raising and capacity-building in order to increase participation, improve decision-making power, and generate action in the health sphere (36, 37). Pederson et al. (38) developed a model representing a continuum of potential gender-responsive interventions derived from discussions during the HIV-AIDS epidemic (39) and emerging evidence on factors relating to how health interventions apply to gender (9) (Figure 1). This continuum, based on the WHO's Gender Responsive Assessment Scale (40), illustrates that health interventions can exploit, accommodate, or transform gender norms, systems and relations in the way that they frame an issue, use imagery and language and/or engage with gender inequity. A gender-sensitive approach is the first, crucial step toward a gender-responsive policy, as it indicates awareness of gender inequalities. However, it does not address these inequalities. Gender-specific and gender-transformative approaches to health interventions are essential, progressive steps toward gender mainstreaming.

Gender-transformative approaches “actively strive to examine, question, and change rigid gender norms and imbalance of power as a means of reaching health as well as gender equity objectives” [(41), p. 8, (40)]. This includes the development of approaches that avoid reproducing harmful gender norms or stereotypes, and empower men and women to attain their health potential. Gender-transformative approaches are characterized by four core principles (38). These interventions are women-centered hereby empowering and considering women as active agents of change in their lives. Interventions that foster harm-reducing approaches provide pragmatic support by helping with immediate goals and providing a variety of options and support. They focus not only on narrow goals related to change in specific health behaviors, but also on facilitating change across the full range of influences and harms associated with this behavior (42). Also, interventions that are explicitly trauma-informed through for example reparative, trustworthy relationships (43) are promising ways of engaging directly with how gender shapes women's health. Finally, gender-transformative interventions are strengths-based and focus on restoring and building health, rather than identifying women's shortcomings (44).

Evidence indicates that programmes incorporating a gender-transformative approach and promoting gender-equitable relationships between men and women are more effective in producing behavior-change than gender-sensitive or gender-neutral programmes (45). This gives weight to the argument that multi-issue programmes using a more nuanced social constructionist and ecological framework are more effective than single issue and individual-focused interventions. Thus, integrated programmes are most effective in changing behavior (45). Heymann et al. (46) define multisectoral action, multilevel and multistakeholder involvement, and diversified programming as key features of high-quality gender-transformative programmes, along with social participation and empowerment. Empowering people to be active agents in their own lives is crucial, but targeting societal structures is equally essential to leverage individual changes and achieve long-lasting effects (47). Programmes that advocate critical reflection among men and women on their socialization into specific gender roles and norms, and even more, that seek to transform norms, have the potential to improve health and wellbeing across many areas of health, and for long periods across the life course (48–50).

The social constructionist approach indicates that gender norms are socially constructed, vary across historical and local contexts, interact with other factors (e.g., poverty and globalization), and are created, reinforced and reconstructed by families, communities and social institutions (51, 52). Gender norms and the reproduction of these norms, are directly related to women's and men's health-related behaviors, with implications for themselves, their partners and their children (53). Over the past three decades, more importance has been placed on treating of “men as partners” in women's health and gender-transformative programming (54). Consequently, a number of international organizations have affirmed the need to engage men and boys in questioning inequitable gender norms (45). Liberating men from gender and sexuality norms that have a negative impact appears to be a decisive step toward attaining gender equity and enhancing health for both men and women (48). Recent research on the implementation of gender-transformative interventions shows that men's involvement is important. For example, a lack of participation of men in programs improving sexual and reproductive health has been revealed as one of the reasons for poor progress in family planning and the use of contraceptives (55). Others show that the engagement of men in family health can create better health (56).

While, a significant body of research found that most organizations have adopted gender mainstreaming policies, little is known about the full effect of these programmes because of several gaps in programme design, implementation and evaluation (57–59). Despite a considerable body of evidence and lessons learned from experiences in applying gender mainstreaming policies, this evidence is limited to certain health areas, such as sexual and reproductive health and rights, newborn, child and adolescent health, HIV/AIDS, and gender-based violence programming and service delivery, with major gaps in other areas with large burdens of disease, including mental health (60). Recently, the United Nations University International Institute for Global Health (UNU-IIGH) and the WHO recognized the urgent need to analyse, extend and transfer lessons learned regarding gender-transformative approaches to other areas of health (61).

The present study was undertaken as part of the GEN-STAR study (GENder-Sensitive Treatment and prevention services for Alcohol and drug useRs) in Belgium, which aimed to assess the availability of, and need for, prevention and treatment approaches sensitive to the needs of women, as well as the obstacles and challenges experienced by female substance users in utilizing these services (62). The findings show that the gender dimension is an actual concern among some alcohol and drug prevention and treatment services in Belgium, and different types of outpatient and residential initiatives sensitive to the needs of women are identified. Still, service users, as well as treatment providers, report a lack of initiatives that are sensitive to the needs of female users (19). Additionally, the current literature in the field of alcohol and drug prevention and treatment focuses mainly on gender-sensitive and gender-specific interventions, while the field of global health promotion pledges that there is more to discover and introduces a gender-transformative perspective (38). The purpose of this study is to explore in-depth how alcohol and drug treatment can be made more sensitive to female users' treatment needs from the perspective of service providers. Consequently, study findings can inform the development of gender-responsive treatment options and aid to a deeper understanding of how these trends are designated on the continuum of approaches to action and health in the alcohol and drug field.

## Methods

### Participants

**Figure 2:**
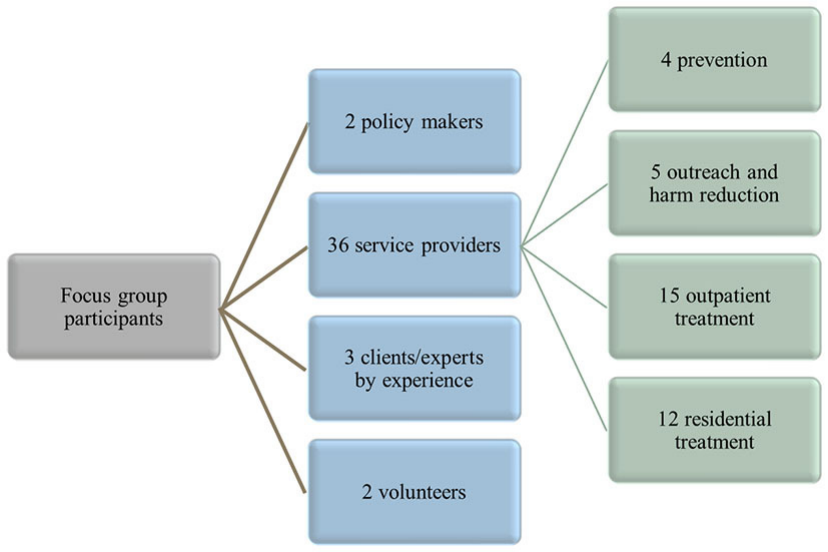
Participants of the four focus groups in Belgium (*n* = 43).

By means of a focus group strategy, prerequisites for implementing services sensitive to women's needs were explored. In total, four focus groups of 8–14 participants were organized across different regions in Belgium, i.e., one in Wallonia, one in Brussels and two in Flanders (*n* = 43). Various stakeholders were involved that are familiar with challenges and obstacles encountered by women in relation to treatment. Alcohol and drug services that were identified in an earlier stage of the research were contacted and invited to participate. Other stakeholders working in the field of alcohol and drug prevention and treatment or who are frequently in contact with women who use drugs were invited as well. The continuum of care was taken into account and reflected in the variety of participants, including social workers, general practitioners, psychologists, nurses, midwifes, and programme coordinators. Additional attention was given to the diversity of profiles by involving men as well as women, younger and more experienced persons, and professional experts as well as former users working as experts by experience, and service users (Figure 2).

### Data collection and analysis

The focus groups were organized between February and April 2018. Each session took approx. two hours and was facilitated by one of the two project researchers, assisted by an additional researcher who took notes throughout the whole process. The GPS Brainstorm toolkit^1^, was used as a structuring method to organize the focus groups and to formulate concrete recommendations for the development and implementation of a more gender-responsive approach in treatment settings for female substance users. This GPS Brainstorm technology was developed by Flanders DC, the Flemish organization for entrepreneurial creativity, and can be used to brainstorm in a group of 8–14 persons. Each group started by stating: “How can we make alcohol and drug services more sensitive to the needs of female substance users?”. To this end, five topics were put forward for discussion, in addition to one empty field. These five specific issues were repeatedly mentioned by female substance users during sixty in-depth interviews in an earlier stage of the research (62): (a) social stigma hindering recognition of specific female needs; (b) responsibilities of women in society as an impediment to self-care; (c) “feeling safe” as a crucial factor in treatment; (d) a plea for a holistic approach; (e) experts by experience and peers to comfort support in treatment. A focus group based on the GPS Brainstorm toolkit consists of three steps: *Generating and classifying ideas*. Participants sit in pairs to explore each specific topic. A board is installed on a table and divided in six parts, each part for one topic. Participants stay around this board. During the session, the GPS board turns and all participants have the opportunity to write ideas on post-its for each theme; *Selecting ideas*. After this first step, participants explore the different ideas on the post-its and vote for ideas that they would like to be realized or that they find particularly pertinent to develop a gender-sensitive approach in the alcohol and drug demand reduction field; *Elaborating ideas in specific project cards*. After having established a ranking of the most relevant ideas, participants briefly develop a project card for the three selected top ideas, including the following elements: title of the idea, definition of the idea, advantages, disadvantages, solutions, impact, and required parties. In the two focus groups in Flanders, the last phase of the brainstorm was not completed due to time restrictions. However, the main ideas were ranked according to relevance, as well as elaborated orally rather than written down on the provided project cards. All the ideas were kept and organized afterwards. Similarities and differences between the different focus groups, as well as the most frequently selected ideas, were discussed among the two project researchers.

## Results

**Figure 3:**
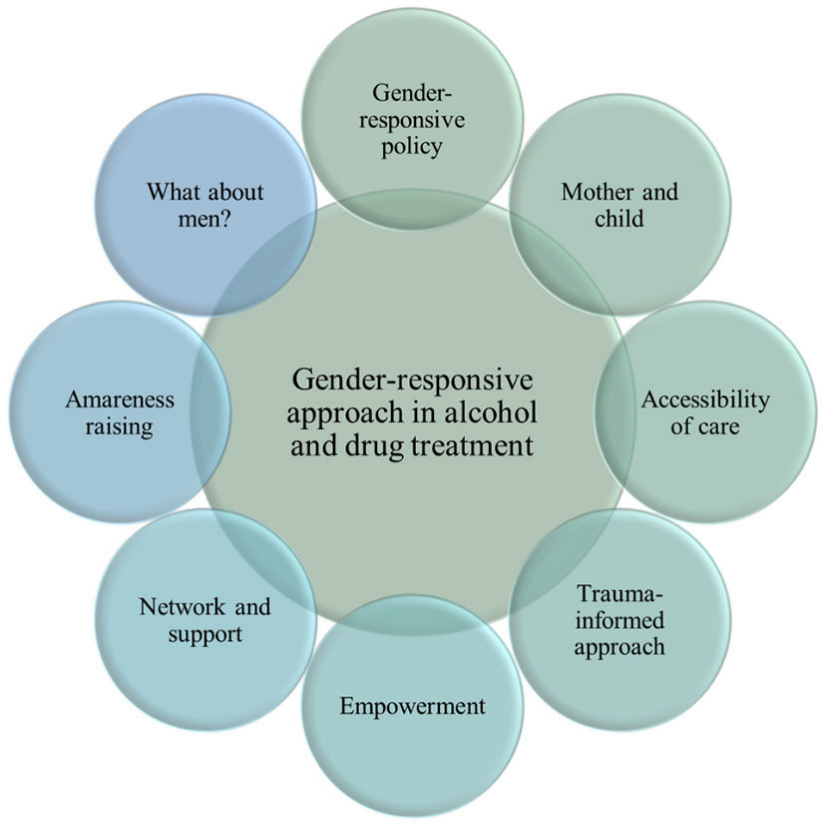
Clusters of themes and interventions toward a gender-responsive approach in alcohol and drug treatment for women.

The focus groups demonstrate different measures and opportunities regarding the implementation of a gender-responsive approach in the alcohol and drug demand reduction field, which are clustered in eight themes (Figure 3). Only the items that were central to participants' experiences are described. The results exclusively reflect the thoughts and ideas of the experts present in the focus groups, in order to improve gender-responsiveness and implement more effective initiatives for women in Belgium.

### Gender-responsive policy

#### Service policy on gender-responsiveness

In pursuit of an optimal integration of gender-responsive measures, several professionals mention the need for a comprehensive policy regarding gender-responsiveness by alcohol and drug prevention and treatment services. This policy is managed in a dynamic, evolving and interactive document available for all staff members. A general philosophy and framework should be included as well as specific guidelines, procedures and practices grafted on the particularities of the service. Hence, this document could serve as a starting point for concrete methods to work with female users in the programme(s) and service(s). In this way, a better understanding of the concept gender-responsiveness can be created, consequently allowing an easier translation to methods and practices.

#### Gender-responsive way of thinking

Three of the focus groups acknowledge that it is essential that all gender-responsive initiatives, measures and programmes are embedded in a global gender-responsive atmosphere, respecting some key features, such as a safe and welcoming environment, accessibility and affordability, and guaranteed anonymity. Further, to ensure continuity and a relationship of trust with female users, initiatives should strive for long-term goals rather than applying short-term projects, and the attitude of staff members working with female users needs to be open-minded and non-judgmental. Further, it is preferable that gynecologists, psychologists or doctors for female users in treatment centers are women, since women tend to talk more openly about sensitive topics with other women than with men. A policy in which a “person of reference” is appointed in treatment centers allows female users to at least have the choice whether their counselor is a man or a woman.

#### Expertise

In all focus groups, the current lack of, and need for, exchanging good practices between professionals is mentioned. Learning from methods and procedures in other programmes is extremely valuable and may offer opportunities for implementation in one's own programme. It can also facilitate collaboration with other alcohol and drug services or cross-system collaboration. A digital platform could meet these needs. In addition, several experts express the need for more training opportunities regarding gender issues. Specific training could be helpful for professionals in order to enhance awareness of women and female users' specificities, as well as existing representations and stigmatizations regarding women and substance use.

### Mother and child

#### Child-friendly setting

One of the most important challenges defined in outpatient services is the lack of practical and infrastructural measures specifically targeting children of mothers who use drugs. Examples are the creation of a kids corner or a room with toys for children, as well as assigning a staff member time to take care of children whilst their mother can have a consultation with a counselor or doctor. But even beyond that, it concerns efforts made toward women to ensure a welcoming place and a service that is well-adapted to their realities. The service should allow more flexibility regarding opening hours and consultations for female users, taking into account their responsibilities as a mother which often hamper help-seeking behavior and treatment retention.

#### Treatment options for substance using mothers and their child(ren)

There are currently eight outpatient treatment services in Belgium for parents/mothers and their child(ren) which are addressed by a great number of female users. However, the demand is higher than the capacity of these programmes, confronting some women with a waiting list and no proper care immediately available. Moreover, the scope of these services is particularly local, leading to counselors not being able to meet the needs of women living further away from services. Regarding residential treatment services for parents/mothers and their child(ren), experts acknowledge a similar challenge. The request for these services is high, whereas the available places are limited, often resulting in a waiting list for mothers in need. Hence, there is a clear demand for more outpatient and residential services for mothers and their child(ren). Related to the focus on families in recovery, maintaining the “kangaroo room” within hospitals is reported to be an issue as well. These specific rooms allow to develop and strengthen the mother-child bond after birth.

#### Detox department reserved for mothers and their child(ren)

In that regard, placement of the child(ren) by child protection services can be avoided as well as a breakdown of the mother-child bond, as this seems to be a major source of motivation for drop-out or continuation of the treatment programme. However, the stakeholders call attention to the fact that this kind of service is promising, but might not be suitable for every mother. Above all, enrolling into treatment as well as agreeing to the conditions related to the treatment setting, will always remain the mother's own choice. The personal situation, the age of the child(ren), the motivation as well as the support of family and friends are all decisive aspects in this context.

#### Multi-sectoral action

Study findings suggest the creation of services that are mainly dedicated to the daily responsibility of a mother toward her child(ren) outside the alcohol and drug demand reduction field. By portraying specific needs and demands regarding child(ren), services are allowed to set up a better and more customized support and assistance not only for mothers who use drugs, but also for their environment. Suggestions for adapted services within this domain of wellbeing for both mother and child(ren) are nurseries, medical care for mothers and child(ren), and parenthood counseling. The idea is to create a supportive structure outside the alcohol and drug demand reduction field, thereby attempting to remove the potential label of “addict,” perceived as an obstacle to care by mothers who use drugs and by society.

### Accessibility of care

#### Alternate services

To optimize accessibility of treatment services, several professionals discuss less conservative and more innovative options to reach female users, such as online self-help groups and a helpline with flexible hours. Women might appeal to these initiatives at the most convenient time for them and from wherever they are. Hence, having children or a lack of transportation would then be less of a barrier to seek treatment. Also, by safeguarding their anonymity, these opportunities reduce the risk of being stigmatized along with feelings of fear and shame. Female users in outpatient settings tend to have more immediate and basic needs (e.g., eating, getting some rest, paraphernalia for injecting drugs, and having a shower). These needs highlight the importance of outreach and street work, like a mobile bus with medical services. The “médibus” in Brussels run by Dune and Doctors of the World is a good practice mentioned in this regard. The staff provides paramedical care, syringe exchange and attempts to implement a harm reduction philosophy. Being available and present at the time that women formulate their need for help, is of crucial value to female users.

#### Financial reimbursement

In all focus groups the experts mark an important lack of easy access to specific services for women who use drugs, like gynecologist consultations or contraception. Often, financial restraints hinder female users to see a doctor or gynecologist, let alone use an appropriate method of contraception. They plea for a minimum price for contraception and health practitioner visits, or even make it costless for vulnerable populations.

### Trauma-informed approach

#### Mapping specific needs

Several professionals indicate the importance of acknowledging specific female needs from the start of the treatment programme in outpatient as well as residential settings. Sensitive topics for women such as trauma, violence, sexual abuse, should be explored and recognized from the intake procedure onwards, and taken into account throughout the entire trajectory of care. In this regard, trauma-informed care does not require trauma treatment within women's substance use treatment, but should instead be seen as a way of working with women likely to have trauma histories that does not retraumatize. Trauma-informed care should be tailored to women's own pace and specific needs. The introduction of trauma specific services can therefore be offered in a staged way (e.g., in aftercare).

#### Combination of single-gender and mixed-gender approaches in treatment

In all focus groups experts call for a combination of single-gender and mixed-gender elements in treatment. From their experiences, women tend to talk easier and more freely in a women-only group. Also, it seems that the absence of men enhances their feelings of safety. On the other hand, the experts acknowledge that women need to learn again to be confronted with men and live together in a healthy way. Treatment including counseling and a safe environment is an ideal way to start this process. The concept of specific support groups for women only in outpatient settings and in residential programmes already exists, but deserves more attention, especially in mixed-gender programmes. These groups allow women to get out of seclusion by creating and maintaining ties, and therefore reinforce their female identity and empowerment. A non-judgmental outpatient initiative where women can be heard about specific women-related themes such as sexuality, sex work, parenthood and violence, and where they can freely share their own reality in a safe environment, can also offer an opportunity to learn more about treatment options in specialized addiction services. These groups can be a safe place to exchange and share knowledge and experiences, besides the added value of talking about sensitive topics. As mentioned before, participants report that the social dynamic differs when an initiative involves both men and women. In that respect, a space dedicated to women only, such as a separate bathroom or a specific living room, is highly recommended in both outpatient and residential mixed-gender services. The dependency on men, and more precisely a man from the close network of a female user, was regularly commented upon by the participants. Hence, the importance of preserving women-only residential programmes is underlined by the stakeholders. These centers allow a safe and welcoming space for women, in which they can focus on themselves, without the pressure of a partner or member of their close network. Nevertheless, in order to maintain a good balance in the diversity of approach and treatment, it is essential to keep the overall composition of staff mixed-gender. Moreover, given the heterogeneity within female substance users, it is crucial to maintain diversity regarding age, sex, ethnicity and professional background in a multidisciplinary team.

### Empowerment

#### Seminars and training

One prominent factor in treatment of female users that was cited frequently is providing a series of psycho-educational seminars and training opportunities. The experts point at some crucial topics that are greatly valuable to focus on in treatment such as self-awareness, self-confidence and self-knowledge. In addition, treatment programmes should offer assertiveness training to female users, empowering them in the strength to stand up for themselves and the courage to refrain from certain situations or people (i.e., often men).

#### Holistic approach

In all focus groups the idea of a holistic approach of care and services is mentioned. In order to consider body and mind in a gender-sensitive approach, it is important that female users can first be seen as women, before being seen as substance users. However, in a holistic perspective not only education or psychotherapy regarding body image is an added value for treatment of female users. Additionally, attention must be drawn to spiritual health. Recovering in a holistic way is healing one's relationships and environment, respecting one's body, setting new perspectives and goals, and finding one's soul purpose. Finding more time and space to learn the pleasures of life again is an integral part of the process. In this regard, experts propose to integrate yoga classes, relaxation sessions and mindfulness sessions in treatment programmes for female users.

### Network and support

#### Professional and private network

Experts suggest the need to create and develop networks around female users, consisting of both professionals and private persons. In regards to the establishment of a professional network, creating a solid network in the field of alcohol and drug demand reduction is essential. However, the professional network should be extended to services not directly related to substance use. Consequently, at the time of admission in treatment every specificity needs to be considered, i.e., issues like mental health, childcare, social support, sexuality, trauma, and housing require special attention. This allows other services to be integrated in women's trajectories of care, for instance child protection services, sex worker services or general healthcare services like a dentist, oculist and gynecologist. Integrated and regrouped services at proximity can facilitate treatment and health care engagement by reducing travel efforts and avoiding losing women between services. However, while support needs to be offered to women in a range of areas, it is important that women be supported to choose the services they are ready to access and be encouraged to pace themselves. A better exchange of information between services is key to a better understanding of each specific situation and ensures better outcomes. To this end, a solid collaboration structure is required. A global socio-medical file could be helpful in creating integrated services and serve as a tool to share information with the different stakeholders. However, special attention must be paid to the informed consent of the client. This is essential as the client is to decide which type of information can be shared between professionals.

#### Experts by experience

Professionals in all focus groups indicate the importance of the active role of female experts by experience in the process of recovery among female users. Such active role could be established in many ways, going from an expert by experience telling her story a single time, to experts by experience working as qualified counselors in the treatment programme. Female users report to feel understood in a better way by experts by experience than by counselors for the reason that the former have experienced a comparable battle, emotions and wheel of life. Further, the participants describe the request of some experts by experience to give significance to their past of substance use by using their experiences to help other female users in their process of recovery. They also express the value of motivating female users to use and share their experiences in the future. The volunteering of experts by experience in a treatment programme is already a point of attention for some centers. However, the lack of time or sufficient staff is problematic to appropriately support experts by experience. Additionally, experts note the concern of anonymity. Female users refuse to talk about sensitive topics and their emotions with someone they formerly know from their drug network. Hence, when working with experts by experience the programme needs to be sure that former users and female users in the programme do not know each other. Also, former users working as experts by experience need to be recognized in their mandate by clients on the one hand and by fellow staff members on the other hand in the pursuit of equivalence among all staff members. Considering these remarks, the stakeholders state that integrating former female users as experts by experience in treatment programmes can be constructive, provided that attention is given to establishing a clear vision and monitoring its implementation. A good programme structure to manage experts by experience is necessary, as well as recognition with regard to mandate and remuneration. Special attention must be given to not replacing the work of staff by experts by experience only, and integrating experts by experience from outside the regional area. Finally, experts propose to develop a network of experts by experience in Belgium. By doing so, a service from one region could consult an expert by experience from another region.

### Awareness raising

#### Prevention campaigns

In all focus groups the importance of campaigns for prevention and awareness raising concerning women and substance use targeted at female users themselves, but also at the societal level is stressed. The social stigma on female users is particularly high and several misconceptions are persistently present in society, resulting in feelings of shame and guilt reported by female users. Whether to focus in these information campaigns on women only or including men and women, should be well-considered. Campaigns should be gender-specific in their design and message. Finally, prevention needs to be innovative in the methods that are used, for example, by looking for opportunities in new technologies, like smartphone apps and social media.

#### General population

First, interventions aimed at reducing the stigma on mental illness and substance use, especially regarding female clients, are desirable. The Flemish mental health prevention campaign “Te Gek” is mentioned as an example of good practice and a starting point to develop new prevention campaigns. A campaign of this nature could raise awareness about gender stereotypes, inequalities and attitudes among a broad audience and could result in a more gender-sensitive attitude toward vulnerable groups. In general, public education and information campaigns are not sufficient for reducing alcohol-related problems. Media advocacy approaches, however, can be helpful to gain public support for policy changes.

#### Professionals

Second, prevention should target professionals supporting female users (such as general practitioners, gynecologists, dentists, counselors) to make them aware of the specific needs of this population and to decrease the judgements that female users might be exposed too. Such judgements could again induce feelings of shame and guilt, which is counterproductive for seeking help. To increase efficient referrals, adequate information on helping resources for female users and their families is necessary for general health and mental health professionals.

#### Women who use drugs

Third, prevention campaigns regarding the use of contraception and other aspects of female hygiene should be targeted specifically to female users. Harm reduction messages concerning, for example, infectious diseases and the use of legal and illegal substances could prevent harm on various health domains. Based on the epidemiological findings, it is clear that gender-specific information should at least include information on alcohol and medication, since these are the main substances for female users.

#### Treatment centers

In all focus groups a global policy of awareness-raising on stereotype gender roles, attitudes, behaviors and inequalities within the centers and more globally in society was deemed an important focus. This type of initiative requires including men imperatively, and holding a multi-foci perspective, allowing various themes such as domestic violence, parenthood, familial responsibilities and contraception to be discussed and worked through. For the stakeholders, these themes are directly linked to the multiple roles and responsibilities that women face in their everyday life and that entail a permanent social pressure. Consequently, providing services and facilitating treatment entry for women is complicated. It is essential to create separate group sessions for both men and women, in addition to mixed-gender therapy groups and comprehensive psycho-educational seminars within treatment centers. Awareness among both men and women needs to be raised on the different roles that women take up in society. Doing so in treatment, allows women to be better prepared for a life after treatment on the one hand, and opens up the possibility to break through stereotyped thinking. Another way to reduce stigmatization and stereotype gender attitudes can be by implementing gender-challenging activities in treatment centers. These activities contain inversed or neutral roles, for example a mechanic class for women and a knitting course for men. Residents in the treatment programme can choose the activity of their preference.

### What about men?

An overall remark and topic that is recurrently discussed is the role of men in the narratives of female users. The experts in the focus groups indicate that men have a responsibility toward women and many related issues such as gender stereotypes and responsibilities in society, as well as the social stigma on female users. In many of the above mentioned ideas and points of attention, men need to be involved and addressed as much as women. Prevention and awareness raising campaigns not only have to address and inform women, but also need to make men aware of certain historically grown societal norms and tendencies influencing women and the way they are looked upon. Also, men need to be involved in the use of contraception and the accessibility of a general practitioner and a gynecologist in order to encourage and manage birth control and sexual health among women. Further, when developing and implementing psycho-educational seminars in treatment programmes around topics such as assertiveness, body image and responsibilities, men need to be involved in a certain way and at a certain time in the process. Though in doing so, counselors need to be cautious to still allow women to feel safe at any time.

## Discussion

Extensive international research has shown that female substance users experience several internal and external barriers to treatment (i.a., 17, 34). In addition, service providers, as well as service users, report a lack of alcohol and drug treatment initiatives in Belgium that are sensitive to the needs of women (19). This is one of the first studies internationally to explore in depth how policy, service providers and service users jointly evaluate and assess the field of alcohol and drug prevention and treatment, and debate on the way forward, i.e., the nature, degree and quality of gender-responsiveness that is needed within programmes or policy in alcohol and drug treatment services.

The perspective of the participants on substance use prevention and treatment for female users incorporates some crucial gender-specific and gender-transformative features. In line with previous research identifying multisectoral action and multistakeholder involvement as crucial features of a gender-transformative approach (45, 46), this study reinforces the need for an integrated approach to support that is sensitive to the needs of female substance users. Findings demonstrate that an integrated approach to the gender dimension is needed, i.e., a strategy that aims to strengthen gender equity in society by giving the gender dimension a place in the content of policies throughout the continuum of care, e.g., not only in alcohol and drug treatment services, but also in prevention and health promotion, and general health services. In order to make care more attuned to female users, adopting gender mainstreaming policies in a diversity of services and organizations is essential to complement more concrete actions at the structural and individual level, such as empowering female substance users and providing services for women (63).

Further, this study shows a lack of approaches that are sensitive to the needs of women in substance use prevention in Belgium and a need for integrated prevention campaigns, focusing on awareness raising and sensitization. These initiatives should not only target girls and young women, but society at large (64, 65). Also, prevention initiatives should be embedded in a broader long-term approach of substance abuse prevention and treatment, rather than serve as a stand-alone campaign (66). Further, the subject of gender-sensitive prevention should not only focus on female substance use, but also on gender inequity, gendered roles and responsibilities in society, and social stigma (67, 68). Consistent with research that has found that diversified programming is key to a gender-transformative approach (46), the present results demonstrate that prevention campaigns must reinforce one another and address the issue of substance abuse from multiple perspectives. Also, by expanding the focus of prevention campaigns to issues broader than substance use itself, alcohol and drug prevention fosters a harm-reducing approach.

Parallel to the findings of prior research (47), our findings indicate that services for alcohol and drug treatment and after care benefit from an integrated approach of support for female substance users. On a more structural level, participants emphasize the importance of building networks of well-cooperating services to support women in a range of areas, such as mental health, child care, education, and housing (63). These general services function best when reinforcing one another, as well as alcohol and drug services, assuring a harm-reducing perspective (69). Rather than isolated gender-responsive services and programmes, the alcohol and drug addiction field should aim at creating full integration of gender expertise and accountability across the sector (61). In keeping with an integrated orientation, the research results also show the need for several specific services for women, i.e., outpatient and residential treatment services for mothers and their child(ren), a detox or crisis intervention center in residential treatment for women and mothers with their children, easy accessible online support, and services for general care and wellbeing of women. The latter in the pursuit of reducing or ideally even defeating the social stigma surrounding female substance use (70, 71). However, participants express the importance of both women-only and mixed-gender aspects in the trajectory of care of female users. Engaging both men and women in questioning inequitable gender norms and empowering women leads to a greater impact on health outcomes (45).

In addition to targeting societal and organizational structures, the study results indicate various adaptations to programmes, interventions and services in order to make them more responsive to the needs of women (72). Women need to be empowered to be active agents in their own lives. The call for psycho-educational seminars on e.g., self-awareness, self-confidence and self-knowledge, and assertiveness training in treatment programmes coincides with the core principles of gender-transformative approaches, i.e. interventions that are explicitly women-centered and strengths-based (38). Further, programmes for female users need to adopt a holistic approach (73), respecting and addressing recovery in a physical, mental and spiritual way.

Last, and recurring throughout all focus groups, is the crucial role of men in the narratives and trajectories of care of female substance users. There is not only a need to learn from and effectively engage with men as they bring new perspectives (61), but also to question and raise awareness among men about historically grown inequitable gender norms and their impact on health and stigma. Men must be involved in changing rigid and harmful gender norms that affect women's health in society and in treatment centers.

In sum, according to the study findings, the alcohol and drug addiction care should seek to move beyond a narrow focus on individual-level change, and equally center on restructuring the power relationships that create and maintain gender inequalities. A significant body of measures mentioned in our research is in line with a gender-transformative approach. Also, the methodology of the research itself (i.e., four heterogeneous focus groups) advocates a gender-transformative approach as a multi-sectoral group including researchers, outpatient as well as residential service providers, prevention workers, mental health clinicians, government policy analysts, service users and experts by experience were united.

This study is the first of its kind in Belgium to investigate the road to a gender-transformative approach in the field of substance use from the perspective of policy, service providers and users. It could serve as basis for further research, in particular to develop a stronger knowledge base around gender-responsive programmes, and to identify the prerequisites for development and implementation of these programmes. To achieve improvements in the lives of both women and men, and generate more equal chances and opportunities in substance abuse treatment, the gender-transformative approach in addiction care needs to be further explored, criticized and established in future research. Drawing on this knowledge, policymakers will be able to develop best practice guidelines that will meet the needs of women. Programme developers and policymakers are tasked with moving our findings from a set of recommendations and interventions to a coherent set of initiatives, policies and programmes that work to strategically enhance women's lives and health. This study also indicates a need for further examination and discussion of the impact of men's involvement on women in treatment and recovery. More interdisciplinary research should be conducted to define how best to improve engaging men in the transformation of social and gender norms and hopefully to define new strategies for improving the health of female users through integrated, holistic interventions. Finally, a critical conceptual note is made on the continuum of approaches on gender and health. Should we aim for gender equity, i.e., everyone gets the support they need, thus producing equity, or rather pursue gender justice, i.e., addressing the underlying causes of inequity (74)?

## Data availability statement

The original contributions presented in the study are included in the article/supplementary materials, further inquiries can be directed to the corresponding author.

## Ethics statement

The studies involving human participants were reviewed and approved by the Ethical Committee Faculty of Psychology and Educational Sciences University of Ghent. The patients/participants provided their written informed consent to participate in this study.

## Author contributions

JS and WV conceived and designed the study. JS conducted the focus groups, led the manuscript writing, and incorporation of the focus group results. WV and FM reviewed the manuscript. All authors read and approved the final manuscript.

## Funding

This work was supported by the Belgian Science Policy (DR/00/73) and the Federal Public Service Health, Food Chain Safety and Environment.

## Conflict of interest

The authors declare that the research was conducted in the absence of any commercial or financial relationships that could be construed as a potential conflict of interest.

## Publisher's note

All claims expressed in this article are solely those of the authors and do not necessarily represent those of their affiliated organizations, or those of the publisher, the editors and the reviewers. Any product that may be evaluated in this article, or claim that may be made by its manufacturer, is not guaranteed or endorsed by the publisher.

## Footnotes

^1^ https://www.flandersdc.be/en
